# Identification and Allelic Variants Associated With Cold Tolerance of *PmPIAS* in *Pinctada fucata martensii*

**DOI:** 10.3389/fphys.2021.634838

**Published:** 2021-03-02

**Authors:** Zhuoxin Lai, Linda Adzigbli, Qingyue Chen, Ruijuan Hao, Yongshan Liao, Yuewen Deng, Qingheng Wang

**Affiliations:** ^1^Fisheries College, Guangdong Ocean University, Zhanjiang, China; ^2^Development and Research Center for Biological Marine Resources, Southern Marine Science and Engineering Guangdong Laboratory (Zhanjiang), Zhanjiang, China; ^3^Pearl Breeding and Processing Engineering Technology Research Centre of Guangdong Province, Zhanjiang, China; ^4^Guangdong Provincial Engineering Laboratory for Mariculture Organism Breeding, Zhanjiang, China

**Keywords:** *Pinctada fucata martensii*, PIAS, cold tolerance, SNPs, expression pattern

## Abstract

The protein inhibitor of activated STAT (PIAS) functions in diverse aspects, including immune response, cell apoptosis, cell differentiation, and proliferation. In the present study, the *PIAS* in the pearl oyster *Pinctada fucata martensii* was characterized. The sequence features of PmPIAS were similar to that of other PIAS sequences with PIAS typical domains, including SAP, Pro-Ile-Asn-Ile-Thr (PINIT), RLD domain, AD, and S/T-rich region. Homologous analysis showed that PmPIAS protein sequence showed the conserved primary structure compared with other species. Ribbon representation of PIAS protein sequences also showed a conserved structure among species, and the PINIT domain and RLD domain showed the conserved structure compared with the sequence of *Homo sapiens*. The expression pattern of *PmPIAS* in different tissues showed significant high expression in the gonad. *PmPIAS* also exhibited a significantly higher expression in the 1 and 2 days after cold tolerance stress (17°C) and showed its potential in the cold tolerance. The SNP analysis of the exon region of *PmPIAS* obtained 18 SNPs, and among them, 11 SNPs showed significance among different genotypes and alleles between cold tolerance selection line and base stock, which showed their potential in the breeding for cold tolerance traits.

## Introduction

In aquaculture, changes in temperature influence the growth, development, reproductive ability, and survival of organisms (Viergutz et al., 2012; Aagesen and Hase, 2014). The fluctuating temperature could compromise and alter the immune function and cause bacteria to proliferate and accumulate in tissues, leading to diseases and stress (Yu et al., 2009). Pearl oysters as warm-water shellfish species were cultured in natural seas and exposed in diverse temperature ranges and their corresponding effects (Adzigbli et al., 2020b). *Pinctada fucata martensii*, as the main marine pearl called “South China sea pearls” production in China (Yang et al., 2019; Zhang et al., 2021), hold a low enduring capacity to extreme temperatures and just hold an optimal range of 23–28°C (Deng et al., 2010). During pearl culture, the juvenile of pearl oyster is transferred to a culturing site in the sea and thus exposed to various environmental perturbations that affect its survival and growth in the natural sea (Hao et al., 2019; Adzigbli et al., 2020b). The sensitivity of pearl oysters to temperature change has limited their culture area, which in turn affects the pearling industry. Therefore, understanding the response mechanism of the pearl oyster to environmental stress and exploring the genetic mechanism of cold tolerance in pearl oyster are essential for the breeding production.

Recently, multiple researches were performed to explore the mechanism of temperature stress such as *Mytilus galloprovincialis*, *Mytilus trossulus*, and *Crassostrea gigas*, and plenty of genes were identified especially genes like HSPs or HSP-related genes (Ioannou et al., 2009; Fields et al., 2012; Zhu et al., 2016). Through the analysis of high-throughput data, cellular senescence, homeostatic flexibility, inhibition of apoptosis, lysosome protein-encoding genes, and immune-related genes participated in the process of temperature tolerance (Ibarz et al., 2010; Zhu et al., 2016; Wang et al., 2018). Liu et al. (2018) showed that stearoyl-CoA desaturase showed a significantly higher expression in the low temperature tolerance, which showed the potential function of monounsaturated fatty acid in the response of temperature (Liu et al., 2018). Although many genes and related metabolic pathway have been reported to be involved in temperature stress, small genetic markers that are important for breeding were screened.

Pearl oysters rely on their innate immunity in response to diverse stress including cold tolerance and diseases (Adzigbli et al., 2020a). The protein inhibitor of activated signal transducer and activator of transcription (PIAS) is a key small ubiquitin-related modifier protein (SUMO) E3 ligase, which participates not only in cytokines but also in various immune signaling pathways (Shuai and Liu, 2005) and may be involved in diverse immune response to stimuli. In addition, extreme temperatures have been known to influence apoptosis, with PIAS protein performing this function in various organisms. PIAS proteins could modulate the function and localization of many proteins, including many components of two important pathways, the NF-κB and JAK-STAT signaling pathway (Schmidt and Müller, 2002; Shuai and Liu, 2003). Furthermore, diverse functions have been ascribed to PIAS in vertebrates and invertebrates including immune response, cell apoptosis, cell differentiation, and proliferation (Duval et al., 2003; Myllymäki and Rämet, 2014). Duck PIAS2 could interact with duck interferon regulatory factor 7 and inhibits IFN-β promoter activation induced by the duck IRF7 (Zu et al., 2020). Amphioxus PIAS inhibits NF-κB by binding with upstream signaling adaptor TICAM-like and MyD88 (Wang et al., 2017). Although most of the PIAS research has focused on model species like Drosophila and mammals, some information is available for aquatic organisms like shrimp *Litopenaeus vannamei* (Zhang et al., 2019). In the present study, the E3 SUMO-protein ligase PIAS in the pearl oyster *P. f. martensii*, a bivalve mollusk, was characterized, and its exon region SNPs related to temperature stress were also investigated to explore its potential in the breeding program for the cold tolerance.

## Materials and Methods

### Experimental Samples

In the present study, *P. f. martensii* was sampled and acquired from Xuwen, Zhanjiang, Guangdong Province, China (20°250′ N, 109°570′ E). Six individuals about 1.5 years old with shell length ranging from 5 to 6 cm were utilized for tissue expression pattern including adductor muscle (A), gill (GI), hepatopancreas (HE), mantle (M), foot (F), and gonad (GO). For the expression analysis of *PmPIAS* in cold tolerance, a total of 150 individuals about 1.5 years old with shell length ranging from 5 to 6 cm were collected and cultured. The water temperature was set at 17°C as cold tolerance and 22°C as control group according to the previous research of Liu et al. (2018) and Wang et al. (2018). The salinity was 30‰. During the experiment, the pearl oysters were cultured with the same amounts of single-cell algae. Eight individuals from both groups were randomly obtained at 6 h, 1, 2, 3, 5, 10, and 15 days. GI tissues were collected for further analysis.

### Gene Cloning and Sequence Analysis of *PmPIAS*

A partial sequence of the *PIAS* gene used for cloning was acquired from *P. f. martensii* genome data (Du et al., 2017). RACE technology was utilized for the full length of the *PmPIAS* gene. In order to obtain the template for the nested PCR, the total RNA was extracted with Trizol reagent (Invitrogen). SMART^TM^ RACE cDNA amplification kit was used to prepare 5′ and 3′ RACE templates that were amplified via nested PCR to acquire the full-length sequence of *PmPIAS* with primers in Table 1. The PCR product was detected using 1% agarose electrophoresis. The gene fragments with appropriate length were sequenced, jointed with DNAMAN software, and then analyzed with BLAST^1^. Open reading frame (ORF) was obtained with ORF finder tool^2^. PIAS protein sequence analysis from different species was performed by Clustal omega^3^, SMART^4^, PHYRE2^5^, and Chimera 1.13.1.

**TABLE 1:** Primers used in the present study.

**Primer**	**Primer sequences (5′–3′)**	**Application**
*PmPIAS*-5**′**-outer	CTTTGAACCTGGTCTGAAATCTCTT	5**′**RACE
*PmPIAS*-5**′**-inner	CTGGCTTTCCTGTTGGTTTTCG	5**′**RACE
*PmPIAS*-3**′**-outer	TCTTTCACCTACGATGCCCAAT	3**′**RACE
*PmPIAS*-3**′**-inner	ACAGACATGCTGATCATACACGG	3**′**RACE
*PmPIAS*-F	ATTACTCCAATCCGATGGGTGC	qRT-PCR
*PmPIAS*-R	CTTTGAACCTGGTCTGAAATCTCTT	qRT-PCR
β-Actin-F	CGGTACCACCATGTTCTCAG	qRT-PCR
β-Actin-R	GACCGGATTCATCGTATTCC	qRT-PCR

### *PmPIAS* Expression Pattern in Tissues, Development, and Cold Tolerance

Real-time quantitative PCR (qRT-PCR) analysis was performed with Thermo Scientific DyNAmo Flash SYBR Green qPCR Kit (Thermo Scientific) in Applied Biosystems 7500/7500 Fast Real-Time PCR system (Applied Biosystems, Foster City, CA, United States) to identify the expression pattern of *PmPIAS*. Expression analysis of different tissues was performed. The qRT-PCR program was 95°C for 30 s, followed by 40 cycles of 95°C for 5 s, and 60°C for 30 s according to the manufacturer’s instructions (Lei et al., 2017). Different stages of development from transcriptomes were collected and analyzed for the expression profiles of *PmPIAS* (Du et al., 2017). Expression pattern of eight individuals from different time points after cold tolerance was performed. Primers used in the present experiment are shown in Table 1.

### SNP Screening of *PmPIAS*

The pearl oysters utilized for the SNP screening were sampled from the third line selected for resistance to cold tolerance. In September 2013, pearl oysters were sampled from a base stock farm in Leizhou of Zhanjiang, Guangdong. The oysters were transported to Nan’ao Island of Shantou, Guangdong, with a temperature of 2–3°C lower than that in Leizhou of Zhanjiang, Guangdong, and cultured in the natural sea. The pearl oyster stayed over winter at Nan’ao Island of Shantou, and the endured individuals were utilized to culture the first line in April 2014. The procedures for larval, juvenile, and adult rearing were explained by Deng et al. (2010). In November 2015 and April 2017, low-temperature resistant selection lines F2 and F3 were cultivated in accordance with the same route. In November 2018, adductor muscles of samples were obtained from the low-temperature resistant selection line (R) and the base stock (W) (shell length ranging from 5 to 6 cm). DNAs from W and R were extracted for SNP identification in the exon region of *PmPIAS* with the method of resequencing (Lei et al., 2019; Yang et al., 2020), and the data were deposited in European Variation Archive, accession number PRJEB43188.

### Statistical Analysis

Popgene32 software was used to calculate the number of alleles, the expected heterozygosity (He), the allele frequency, the effective number of alleles (Ne), and the observed heterozygosity (Ho). PIC software was used to calculate the polymorphism information content of the SNP loci (Yang et al., 2020). Statistical differences in SNPs between the R and W were obtained using chi-square test on SPSS 16.0 software (Chicago, IL). The expression of *PmPIAS* at tissues and different time points after cold tolerance was calculated using the 2^–Δ^ ^Ct^ method with β-actin gene as the reference gene and then analyzed by SPSS 16.0. *P* < 0.05 was considered statistically significant.

## Results

### Cloning and Sequence Analysis of *PmPIAS*

**FIGURE 1:**
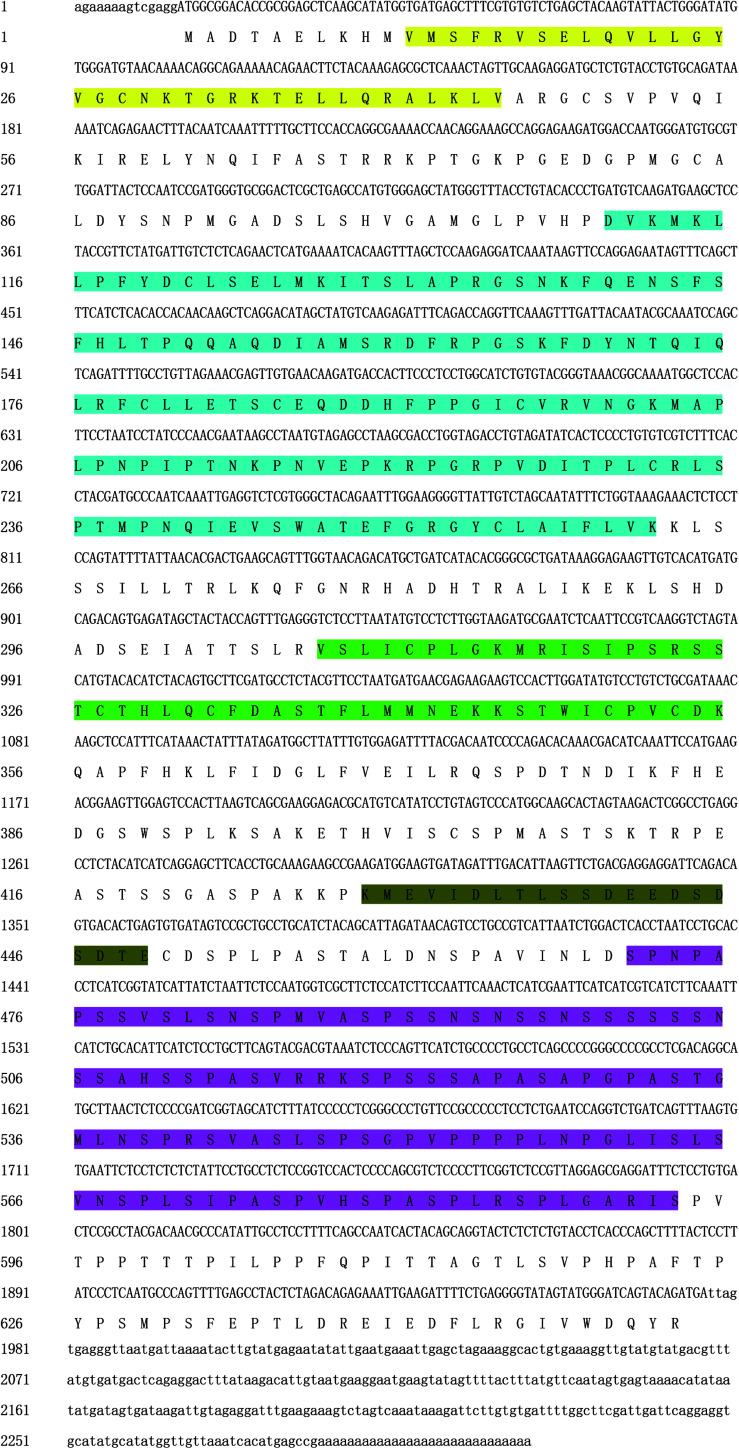
Nucleotide sequence analysis of *PmPIAS*. 5′UTR and 3′UTR are indicated in small letters. ORF and deduced amino acid sequences are indicated in capital letters. Sequence in yellow, light blue, green, dark green, and purple background represents SAP, PINIT, RLD domain, AD, and S/T-rich region, respectively.

The full length of *PmPIAS* was 2,313 bp with 5′UTR of 14 bp and 3′UTR of 337 bp. The ORF of *PmPIAS* was 1,962 bp, encoding 653 amino acids (Figure 1). The deduced aa sequence of PmPIAS featured a theoretical molecular weight of 70.53 kDa. Domain analysis showed that PmPIAS had typical PIAS domains, including SAP, PINIT, RLD domain, AD, and S/T-rich region. The complete sequence of *PmPIAS* was deposited in GenBank under the accession number MW326754.

### Structural and Homologous Analysis

**FIGURE 2:**
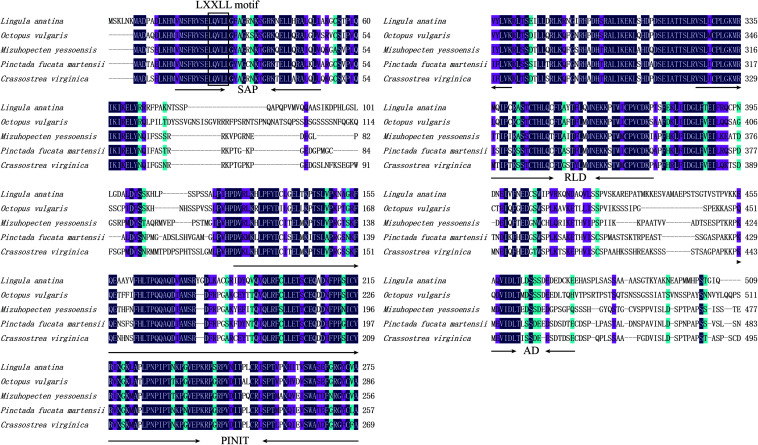
Multiple-sequence alignment and structural organization of PmPIAS aa sequences. Dark blue background indicates conserved aa, pink background indicates aa with strong similarity, light blue indicates aa with weak similarity, and the numbers on the right show the position of sequence alignment. The frame showed the conserved motif (LXXLL). The accession numbers for sequences used in this alignment and structure analysis are as follows: *Crassostrea virginica* (XP_022331287.1), *Mizuhopecten yessoensis* (XP_021340266.1), *Octopus vulgaris* (XP_029634642.1), and *Lingula anatina* (XP_013385044.1).

**FIGURE 3:**
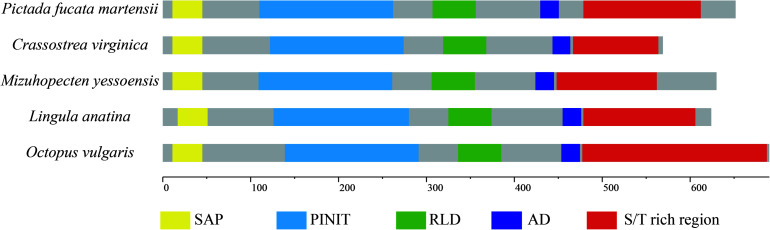
The structural organization analysis of PmPIAS. The frame of yellow, light blue, green, dark blue, and red represents SAP, PINIT, RLD domain, AD, and S/T-rich region, respectively. Accession numbers for sequences used in this alignment and structure analysis are as follows: *Crassostrea virginica* (XP_022331287.1), *Mizuhopecten yessoensis* (XP_021340266.1), *Octopus vulgaris* (XP_029634642.1), and *Lingula anatina* (XP_013385044.1).

Homologous analysis was performed with PIAS sequence from *Crassostrea virginica* (XP_022331287.1), *Mizuhopecten yessoensis* (XP_021340266.1), *Octopus vulgaris* (XP_029634642.1), and *Lingula anatina* (XP_013385044.1) to determine the homology of PmPIAS with other species. The results showed that PmPIAS had the highest identity with *C. virginica* (75.75%) (Figure 2). The PmPIAS hold the identities of 60.67, 64.38, and 61.43%, compared with *M. yessoensis*, *O. vulgaris*, and *L. anatine*, respectively. The predicted structural organization of PIAS was performed among *C. virginica* (XP_022331287.1), *M. yessoensis* (XP_021340266.1), *O. vulgaris* (XP_029634642.1), and *L. anatina* (XP_013385044.1). PmPIAS protein sequence showed the conserved primary structure compared with other species (Figure 3).

### PIAS Advanced Structure Analysis of Different Species

**FIGURE 4:**
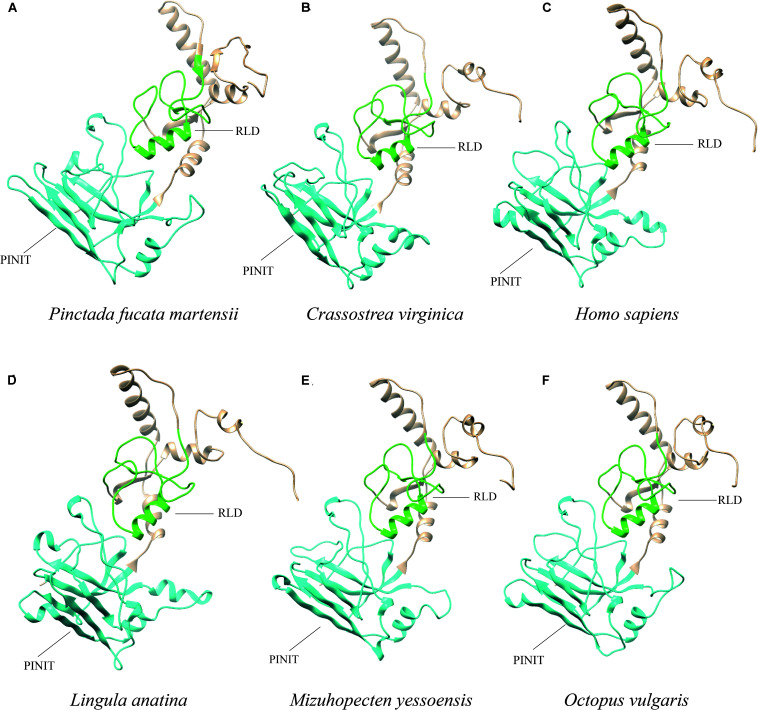
Ribbon representation of PIAS protein sequences from different species. **(A–F)** Show the protein sequences from *P. f. martensii*, *Crassostrea virginica* (XP_022331287.1), *Homo sapiens* (AAI11061.1), *Lingula anatina* (XP_013385044.1), *Mizuhopecten yessoensis* (XP_021340266.1), and *Octopus vulgaris* (XP_029634642.1), respectively. The blue and green represented the PINIT and RLD domains, respectively.

The PIAS protein sequences from *P. f. martensii*, *C. virginica* (XP_022331287.1), *M. yessoensis* (XP_021340266.1), *O. vulgaris* (XP_029634642.1), *L. anatina* (XP_013385044.1), and *H. sapiens* (AAI11061.1) were submitted to Phyre server for homology modeling. Ribbon representation of these protein sequences showed similar structure. Furthermore, the PINIT domain and RLD domain showed the conserved structure compared with the sequence of *Homo sapiens* (Figure 4).

### Expression Analysis of *PmPIAS* in Tissues, Development, and Cold Tolerance

**FIGURE 5:**
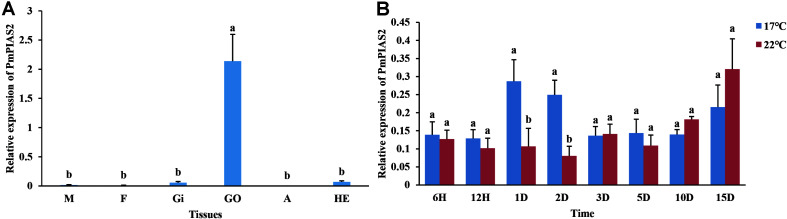
Expression pattern of *PmPIAS* in tissues and cold tolerance. **(A)** Presents the expression pattern of *PmPIAS* in tissues. M, mantle; F, foot; GI, gill; GO, gonad; A, abductor muscle; HE, hepatopancreas. **(B)** Shows the expression pattern of *PmPIAS* under cold tolerance stress (17°C). Columns with different letters are significantly different (*P* < 0.05).

qPCR analysis was conducted to ascertain the *PmPIAS* expression in the various tissues examined (Figure 5A). The result showed that *PmPIAS* hold the significant high expression in GO compared with other tissues. Analysis of the developmental transcriptome of *P. f. martensii* indicated that *PmPIAS* was widely expressed in the development stages and showed high expression in trochophore, followed by fertilized egg and juveniles (Supplementary Figure S1). The temporal expression of *PmPIAS* under cold tolerance stress was examined via qPCR. The most significant high expressions among the different time points were observed at 1 and 2 days (Figure 5B).

### Genetic Polymorphism Analysis of SNPs From *PmPIAS* Exon Region

Eighteen SNP loci were identified in the exon region of *PmPIAS*. The polymorphism information of these SNP loci was analyzed, and the results showed that the PIC ranged from 0.1038 to 0.3749. Among them, nine SNPs with low polymorphism (PIC < 0.25) and nine SNPs with moderate polymorphism (0.25 < PIC < 0.5) were found. Ho ranged from 0.1167 to 0.6000. The range of He is 0.1108–0.3749. HWE analysis indicated that 18 SNP did not deviated from HWE (*P* > 0.05) (Table 2).

**TABLE 2:** Polymorphism analysis of SNPs in *PmPIAS*.

**SNP name**	**Position**	**Ho**	**He**	**PIC**	**HWE**
SNP1	4,900,234 (C > T)	0.4500	0.4167	0.3278	0.5306
SNP2	4,900,243 (C > T)	0.2167	0.2688	0.2310	0.1242
SNP3	4,900,273 (C > T)	0.2167	0.1948	0.1745	0.3677
SNP4	4,900,303 (T > G)	0.5333	0.4952	0.3705	0.5478
SNP5	4,901,462 (G > A)	0.1167	0.1399	0.1291	0.1739
SNP6	4,901,547 (G > A)	0.4500	0.4973	0.3716	0.4571
SNP7	4,902,338 (A > G)	0.1500	0.1399	0.1291	0.5553
SNP8	4,902,368 (C > T)	0.1667	0.1815	0.1638	0.5097
SNP9	4,903,720 (G > A)	0.4167	0.5041	0.3749	0.1756
SNP10	4,903,771 (C > A)	0.1833	0.1679	0.1527	0.4578
SNP11	4,904,485 (T > C)	0.1167	0.1108	0.1038	0.6581
SNP12	4,904,527 (T > C)	0.2667	0.1331	0.2044	0.2503
SNP13	4,904,822 (C > T)	0.3333	0.3227	0.2688	0.7947
SNP14	4,904,900 (C > T)	0.5167	0.4637	0.3541	0.3718
SNP15	4,904,903 (A > G)	0.1167	0.1108	0.1038	0.6581
SNP16	4,910,826 (C > T)	0.5667	0.4768	0.3610	0.1403
SNP17	4,910,841 (T > G)	0.6000	0.4840	0.3648	0.0611
SNP18	4,910,873 (G > A)	0.5667	0.4768	0.3610	0.1403

### SNP Analysis of Genotype and Allele From *PmPIAS* Between R and W

A total of 18 SNP sites were detected in the exon region of *PIAS*, 11 of which significantly differed between W and R (*P* < 0.05). Among them, the frequencies of genotype TT of g.4900234 was 70% in the R, and 26.7% in the W; the genotype CT of g.4900243 in the two lines were 40 and 3.3%, respectively. The frequencies of genotype GG of g.4903720 in the W and R were 50 and 6.67%, respectively. The frequencies of genotype CT of g.4904822 in the W and R were 56.7 and 10%, respectively (Table 3). These results indicated the potential of the 11 SNPs in cold tolerance selection program.

**TABLE 3:** Analysis of *PmPIAS* SNPs in R and W.

**ID**	**Position**	**Genotype**	**Ind. of R**	**Ind. of W**	***P***	**Allele**	**Ind. R**	**Ind. of W**	***P***
SNP1	4,900,234 (C > T)	TT:CC:CT	21:0:9	8:4:18	0.002**	T:C	51:9	34:26	0.001**
SNP2	4,900,243 (C > T)	TT:CC:CT	3:15:12	0:29:1	0.000**	T:C	18:42	1:59	0.000**
SNP3	4,900,273 (C > T)	TT:CT	28:2	19:11	0.01*	T:C	58:2	49:11	0.016*
SNP4	4,900,303 (T > G)	GG:GT:TT	5:18:7	13:14:3	0.059	G:T	28:32	40:20	0.042*
SNP5	4,901,462 (G > A)	GG:AA:AG	24:1:5	28:0:2	0.273	G:A	53:7	58:2	0.163
SNP6	4,901,547 (G > A)	GG:AA:AG	3:13:14	17:0:13	0.000**	G:A	20:40	47:13	0.000**
SNP7	4,902,338 (A > G)	GG:AG	30:0	21:9	0.002**	G:A	60:0	51:9	0.003**
SNP8	4,902,368 (C > T)	TT:CC:CT	1:19:10	0:30:0	0.001**	T:C	12:48	0:60	0.000**
SNP9	4,903,720 (G > A)	GG:AA:AG	15:0:15	2:18:10	0.000**	G:A	45:15	14:46	0.000**
SNP10	4,903,771 (C > A)	AC:CC	4:26	7:23	0.506	A:C	4:56	7:53	0.529
SNP11	4,904,485 (T > C)	TT:CT	25:5	28:2	0.424	T:C	55:5	58:2	0.439
SNP12	4,904,527 (T > C)	TT:CT	25:5	19:11	0.143	T:C	55:5	49:11	0.178
SNP13	4,904,822 (C > T)	TT:CC:CT	2:11:17	0:27:3	0.000**	T:C	21:39	3:57	0.000**
SNP14	4,904,900 (C > T)	TT:CC:CT	2:17:11	4:6:20	0.014*	T:C	15:45	28:32	0.022*
SNP15	4,904,903 (A > G)	AA:AG	25:5	28:2	0.424	A:G	55:5	58:2	0.439
SNP16	4,910,826 (C > T)	TT:CC:CT	7:2:21	13:4:13	0.114	T:C	35:25	39:21	0.573
SNP17	4,910,841 (T > G)	GG:GT:TT	5:23:2	13:13:4	0.03*	G:T	33:27	39:21	0.352
SNP18	4,910,873 (G > A)	GG:AA:AG	2:7:21	4:13:13	0.000**	G:A	25:35	21:39	0.573

P-values are from chi-square test of genotype and allele frequencies in the R and W. *P < 0.05, **P < 0.01.

## Discussion

As a member of the negative regulators of the JAK/STAT signaling pathway, PIAS participates in the regulation of immune responses, which showed its potential in response to cold tolerance. However, in the bivalve, limited reports have been researched about PIAS proteins. In the present study, a *PIAS* gene from *P. f. martensii* was cloned and SNPs in the exon region of *PmPIAS* were obtained to explore the genetic information for breeding.

Researches have reported that the PIAS protein family exhibit high similarity in conserved domains including the conserved RLD, PINIT motif, C-terminal Ser/Thr amino acids enriched region (S/T), AD, and LXXLL signature motif in the SAP domain (Duval et al., 2003; Suzuki et al., 2009). Among them, SAP domain aids the function of the *PIAS* gene, which is associated with sequence- or structure-specific DNA binding (Aravind and Koonin, 2000). The LXXLL signature motif was found to mediate the interactions between nuclear receptors and their coregulators (Glass and Rosenfeld, 2000). RLD domain is required for the SUMO-E3-ligase activity of PIAS proteins and may be involved in the interaction with other proteins (Kotaja et al., 2002). Amphioxus PIAS inhibited NF-κB activation by co-localizing and binding with TRAF6, and the interaction relied on the N-terminal SAP and PINIT domains of PIAS (Fu et al., 2020). PIASy binds to MafA through the SAP domain and negatively regulates the insulin gene promoter through a novel SIM1-dependent mechanism (Onishi and Kataoka, 2019). In the present study, the protein sequence feature of PmPIAS also contained all conserved domains and motifs, which were consistent with PIAS proteins from other species like *Crassostrea virginica*, *Mizuhopecten yessoensis*, *Octopus vulgaris*, *Scylla paramamosain*, and *Lingula anatina* (Shuai, 2006; Huang et al., 2015). Advanced structure analysis of PIAS proteins from *H. sapiens* and bivalve also showed the conserved structure of PINIT and RLD domains in the PmPIAS. Therefore, PmPIAS may be a member of the PIAS protein family.

PIAS protein participates in regulating various immune signaling pathways and immune response to stimuli (Shuai and Liu, 2005). In *L. vannamei*, LvPIAS exhibited an immune response function after bacteria and virus stimulation with a significant expression pattern within 48 h post-stimulation and inhibited the transcriptional activity of LvSTAT, which indicated that there was a feedback loop between LvSTAT and LvPIAS (Zhang et al., 2019). PIAS of *Scylla paramamosain* was involved in the pathogen-resistant activities of mud scab (Huang et al., 2015). Expression pattern of *PmPIAS* in the different tissues and developmental stages indicated that this gene may play an important role in the life process of *P. f. martensii*. Xenopus *PIAS* plays important roles in mesodermal induction and patterning during early frog development (Burn et al., 2011). After cold tolerance, *PmPIAS* expressed significantly highly at the early time points to respond to the stress, which indicated its important function in the cold tolerance stress response. In the Arabidopsis, the ectopic expression of IZ1 (a SIZ/PIAS-Type SUMO E3 Ligase) could also improve the cold tolerance (Li et al., 2020). Researches have reported that, after low temperature tolerance, Pm-SCD, Pm-HK, and PmHSP70 genes showed a significantly higher expression at different time points compared with the control group, which means the pearl oysters utilized different biological processes at different times to respond to the low temperature tolerance (Liu et al., 2018, 2019a,b). Temperature tolerance has been reported to induce the immune system, and transcriptome analysis of low water temperature stress showed that several immune-related genes and pathways were presented in response to exposure to low temperatures in pearl oysters (Wang et al., 2018). Immune-related genes were also upregulated after cold exposure of *Drosophila melanogaster* and suggested that immunomodulation plays an important role in response to cold stress (Zhang et al., 2011; Tusong et al., 2017). Previous studies reported that PIAS could bind to the transcription factor STAT in the cytoplasm and then inhibit the DNA-binding activity with downstream genes especially immune-related genes and pathways in mammals, jawless fishes, and some crustaceans (Kotaja et al., 2002; Niu et al., 2018; Li et al., 2020). Therefore, *PmPIAS* may participate in the cold tolerance through involvement in the immune activity.

In order to explore the potential of *PmPIAS* in the breeding program for the cold tolerance selection, exon region SNPs of *PmPIAS* were identified. Various studies have reported that particular environmental stresses increase SNPs. Different cold tolerances for Nile tilapia strains from Ghana, Egypt, and Ivory Coast suggest the influence of geographic location and natural selection on cold tolerance in tilapia (Sifa et al., 2002). The further the geographic location from the equator, the more cold-tolerant the strain of Nile tilapia. The two sequenced natural populations of bay scallop *Argopecten irradians* presented a substantial difference in T allele frequency implying that the SNP all-53308-760 T/C may have been subjected to natural selection for temperature adaptation, and the higher frequency of T allele in the southern subspecies is the consequence of local adaptation (Du et al., 2014). In the present study, 11 SNP sites were found in the exon region of *PmPIAS*, and they demonstrated significant differences between the R and W in genotype and allele. Accumulating evidence suggested that genetic polymorphisms in the coding regions could affect protein activity or effect of translation (Gottler et al., 2008; Frydenberg et al., 2010). The SNPs detected in the present study may provide a potential site for the cold tolerance selection in the future.

## Conclusion

The full-length characterization of PmPIAS showed its conserved primary and advanced structures in the protein sequence. Expression analysis demonstrated the wide distribution of *PmPIAS* in pearl oyster and showed a significant increase after cold tolerance. Eighteen SNPs were identified in the exon region of *PmPIAS*, and 11 SNPs showed the potential in the cold tolerance breeding of pearl oysters. This study provided a potential molecular marker for the selective breeding of cold tolerance of *P. f. martensii*.

## Data Availability Statement

The sequence information of *PmPIAS* presented in the study are deposited in the GenBank repository, accession number MW326754. The SNP data are deposited in the European Variation Archive, accession number PRJEB43188.

## Author Contributions

RH and QW: conceptualization. ZL: formal analysis and writing–original draft preparation. LA and QC: resources. RH, QW, YL, and YD: writing–review and editing. All authors have read and agreed to the published version of the manuscript.

## Conflict of Interest

The authors declare that the research was conducted in the absence of any commercial or financial relationships that could be construed as a potential conflict of interest.

## Footnotes

1 https://blast.ncbi.nlm.nih.gov/Blast.cgi

2 https://www.ncbi.nlm.nih.gov/orffinder/

3 https://www.ebi.ac.uk/Tools/msa/clustalo/

4 http://smart.embl-heidelberg.de/smart/set_mode.cgi?NORMAL=1

5 http://www.sbg.bio.ic.ac.uk/phyre2/html/page.cgi?id=index
