# Supplementary material for: Identification and Allelic Variants Associated With Cold Tolerance of PmPIAS in Pinctada fucata martensii
Source: Front Physiol. 2021 Mar 2;12:634838. doi: 10.3389/fphys.2021.634838 (PMC7960669; doi:10.3389/fphys.2021.634838)
Supplement: Supplementary file 1 [file Data_Sheet_1.DOCX]

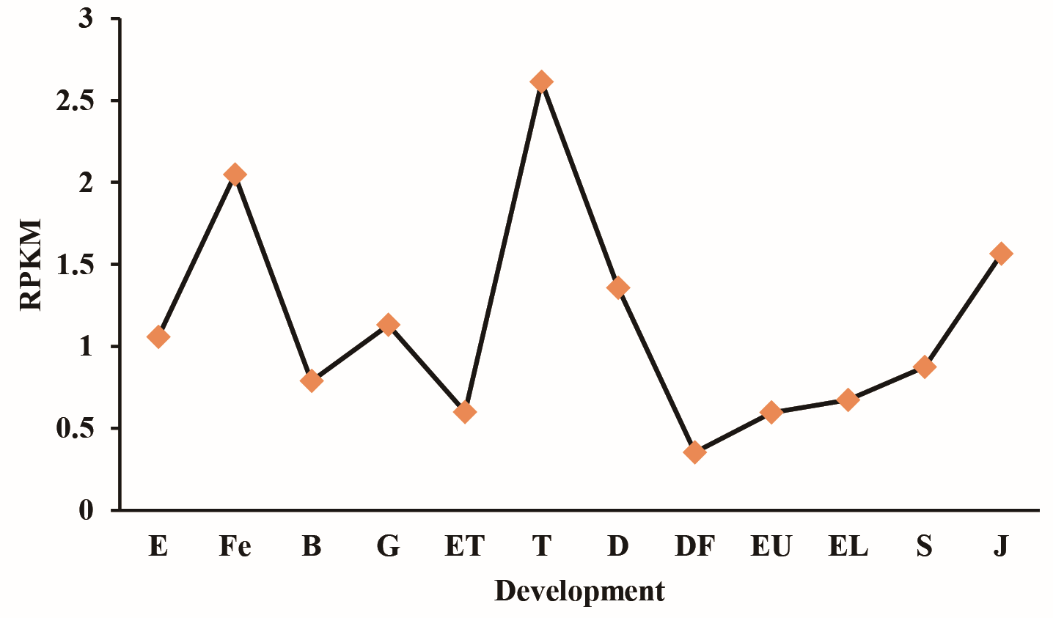


Figure S1 Expression pattern of *PmFGF18* during development in the development transcriptome.

E: egg; Fe: fertilized egg; B: blastula; G: gastrula; ET: early trochophore; T: trochophore; D: D-stage larvae; DF: D-stage larvae before feeding; EU: early umbo larvae; EL: eyed larvae; S: spat; J: juveniles.
