# Supplementary figures and images for: Identification and Allelic Variants Associated With Cold Tolerance of PmPIAS in Pinctada fucata martensii
Source: Front Physiol. 2021 Mar 2;12:634838. doi: 10.3389/fphys.2021.634838 (PMC7960669; doi:10.3389/fphys.2021.634838)

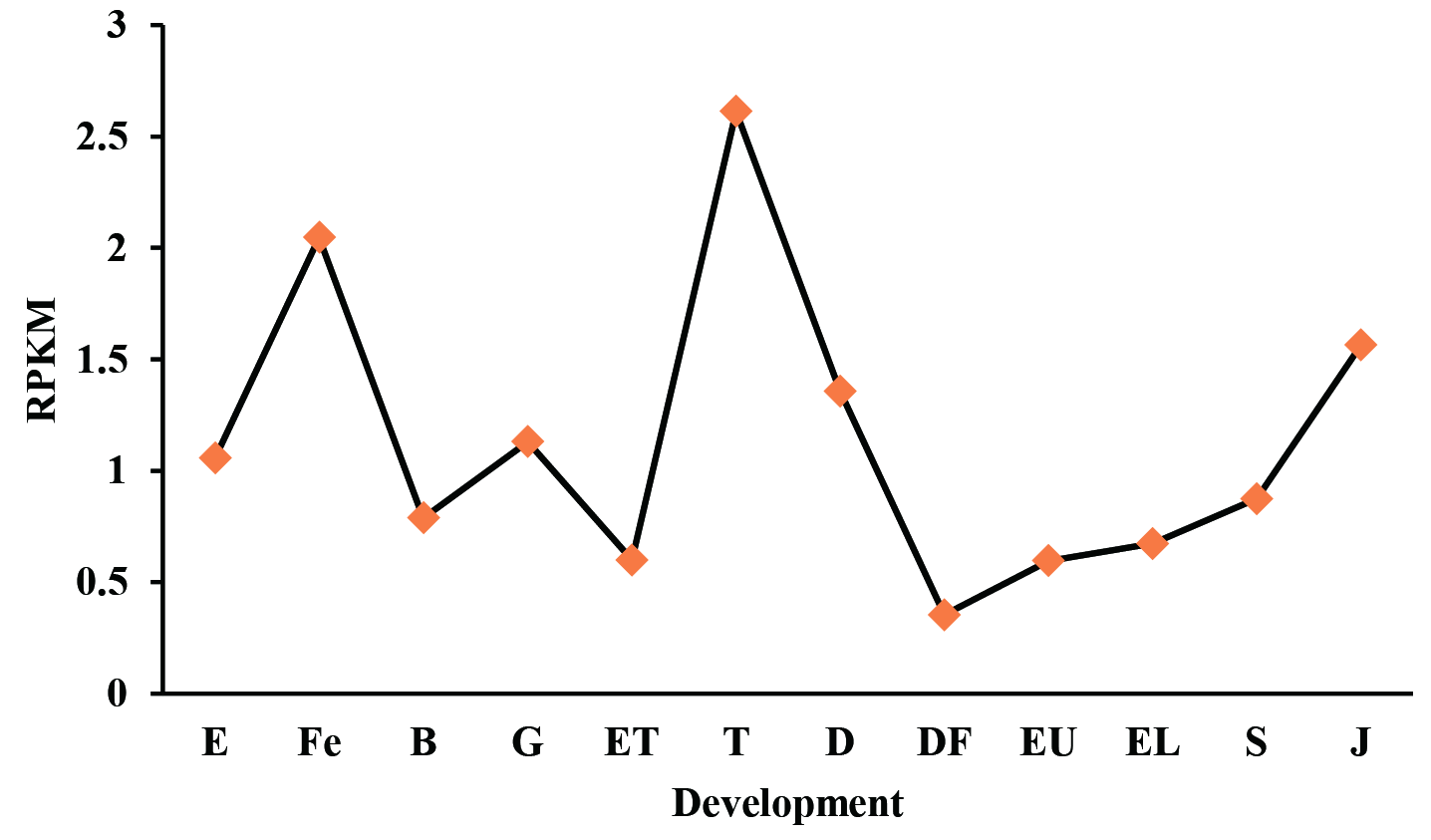

Supplement: Supplementary file 2 [file Image_1.TIF]
